# The Effect of Laminin-1-Doped Nanoroughened Implant Surfaces: Gene Expression and Morphological Evaluation

**DOI:** 10.1155/2012/305638

**Published:** 2012-12-12

**Authors:** Humberto Osvaldo Schwartz-Filho, Kostas Bougas, Paulo G. Coelho, Ying Xue, Mariko Hayashi, Rafael Silveira Faeda, Rosemary Adriana Chiérici Marcantonio, Daisuke Ono, Fumio Kobayashi, Kamal Mustafa, Ann Wennerberg, Ryo Jimbo

**Affiliations:** ^1^Department of Prosthodontics, Faculty of Odontology, Malmö University, 205 06 Malmö, Sweden; ^2^Division of Periodontology, Department of Oral Diagnosis and Surgery, School of Dentistry, UNESP, São Paulo State University, 01049-010 Araraquara, SP, Brazil; ^3^Department of Biomaterials and Biomimetics, New York University, New York, NY 10010, USA; ^4^Department of Clinical Dentistry, Center for Clinical Dental Research, University of Bergen, 5020 Bergen, Norway; ^5^Division of Applied Prosthodontics, Nagasaki University Graduate School of Biomedical Sciences, Nagasaki 852-8102, Japan; ^6^Private Practice, Kobe 658-0012, Japan

## Abstract

*Aim*. This study aimed to observe the morphological and molecular effect of laminin-1 doping to nanostructured implant surfaces in a rabbit model. *Materials and Methods*. Nanostructured implants were coated with laminin-1 (test; dilution, 100 **μ**g/mL) and inserted into the rabbit tibiae. Noncoated implants were used as controls. After 2 weeks of healing, the implants were removed and subjected to morphological analysis using scanning electron microscopy (SEM) and gene expression analysis using the real-time reverse transcriptase-polymerase chain reaction (RT-PCR). *Results*. SEM revealed bony tissue attachment for both control and test implants. Real-time RT-PCR analysis showed that the expression of osteoblast markers RUNX-2, osteocalcin, alkaline phosphatase, and collagen I was higher (1.62-fold, 1.53-fold, 1.97-fold, and 1.04-fold, resp.) for the implants modified by laminin-1 relative to the control. All osteoclast markers investigated in the study presented higher expression on the test implants than controls as follows: tartrate-resistant acid phosphatase (1.67-fold), calcitonin receptor (1.35-fold), and ATPase (1.25-fold). The test implants demonstrated higher expression of inflammatory markers interleukin-10 (1.53-fold) and tumour necrosis factor-**α** (1.61-fold) relative to controls. *Conclusion*. The protein-doped surface showed higher gene expression of typical genes involved in the osseointegration cascade than the control surface.

## 1. Introduction

The osseointegration cascade begins immediately after the implant is placed in the bone, where the blood contiguously interacts with the implant surface. Irrespective of the biomaterial, surface topography, or wettability status of the surface, the initial contact with blood will rapidly attract proteins [1], which in turn will initiate the process of bone formation [2, 3]. In fact, protein adsorption to the implant surface has been suggested to be important for the osteoconduction stage of osseointegration [4–6]. Studies have also investigated the significance of the protein-implant interaction phenomenon [7–9], in which some proteins significantly enhance migration, attachment, proliferation, and differentiation at the implant surface [8]. Protein adsorption to biomaterials is intriguing since one specific protein never remains in a single niche for extended periods of time and constantly undergoes alterations depending on its molecular weight [10]. The so-called “Vroman effect” is an indication that proteins play different roles in biological reactions. For example, the effect of plasma fibronectin has been thoroughly studied along with bone biomechanical properties, and it has been reported to play an important role in the migration and attachment of mesenchymal cells and to be a regulator of bone density [11, 12]. Another example is collagen type I, which is the major constituent protein of the bone matrix, assembles in the presence of fibronectin [13], and is thereby considered indispensible protein for osteogenesis [14]. Hence, intentionally doping implant surfaces with proteins that have direct relationship with osteogenic events such as bone matrix formation may improve both the quality and the quantity of osseointegration [8, 15–18]. 

When protein doping implant surfaces that are typically textured (rough), further topographical alterations may occur and might therefore affect cell adhesion and differentiation by potentially enhancing the effects of adsorbed protein layers [19]. In addition, it has been suggested that substrate surfaces possessing nanostructures show a high affinity for protein adsorption [20–22]. As reported by Puckett et al., intentionally applied nanogrooved surfaces presented higher fibronectin attachment compared to the control surfaces (no grooves) [21]. Such surface topography may be suitable for sustaining higher volumes of target proteins and thus may facilitate implant adherence for a longer duration [8], supposedly owing to the augmented surface area rendered by such length scale texturization. It has been reported that cell morphology, cytoskeleton and adhesion formation, and then subsequent cell growth and differentiation are altered by nanotopographies, thereby stimulating the osteoprogenitor cell differentiation towards an osteoblastic phenotype [23]. These findings are confirmed by another study conducted in human embryonic palatal mesenchymal cells. Apart from alterations in cell morphology, it was demonstrated that an increased gene expression of the osteoblastic markers, Runx-2, and osteocalcin was evident when the cells were cultured on rough and grooved implant surfaces as compared to tissue culture plastic [24].

In the present study, we have focused on a potential osteogenesis-enhancing protein, namely, laminin-1, which is a heterotrimeric glycoprotein that contains an arginine-glycine-aspartic acid (RGD) sequence [25]. RGD is an integrin receptor binder, which is commonly found within the extracellular matrix proteins, and is the most widely occurring cell adhesive motif recognised by about 50% of all known integrins such as *α*_2_ *β*_1_, *α*_3_ *β*_1_, *α*_5_ *β*_1_, *α*_*V*_ *β*_1_, *α*_*V*_ *β*_3_, *α*_*V*_ *β*_5_, and *α*_6_ *β*_4_ [26]. It has been reported that when applied to implant surfaces, the RGD sequence-impregnated modification significantly hastens osseointegration [27, 28] and upregulates the osteoblast focal adhesion through integrin-mediated mechanisms [29]. Besides the well-known bone forming ability of the RGD sequence, another interesting feature of laminin-1 is that it has the ability to selectively recruit osteoprogenitor cells [30]. 

 Another reported feature of the laminin-1 is that it may possibly act as a nucleation center for the precipitation of calcium phosphates (CaP) [31]. It was shown that in a model using the simulated body fluid (SBF), the titanium surface presented more CaP precipitation when laminin-1 was added to the SBF than SBF alone. Thus, it can be hypothesised that this unique protein may have an impact on the initial responses of implant-bone interactions. 

The aim of the present study was to dope an implant surface presenting nanostructures with laminin-1 and observe the biological response at the implant interface. We hypothesised that the addition of laminin-1 would enhance osteogenic markers in the early stages of osseointegration. To test our hypothesis, implants were placed in rabbit tibiae for two weeks; bone morphology and total mRNA were extracted to evaluate the expression of genes involved in the inflammation and bone remodelling processes.

## 2. Materials and Methods

### 2.1. Surface Characterization

Scanning electron microscopy (SEM) (LEO 440-Zeiss, Oberkochen, Germany) was used for the assessment of surface morphology. 

The topography of the control implants was characterized using an interferometer (MicroXam; ADE Phase Shift Technology Inc., Tucson, AZ, USA). The parametric calculation was performed after form errors and waviness were removed with a 50 *μ*m × 50 *μ*m Gaussian filter. The following three-dimensional parameters were selected: *S*_*a*_ (*μ*m), the arithmetic average height deviation from a mean plane; *S*_ds_ (*μ*m^−2^), the density of summits, and *S*_dr_ (%), the developed surface ratio. Three implants were randomly selected for the analysis.

### 2.2. Implants and Laminin Coating

Commercially pure titanium (Grade 4) implants (Neodent, Curitiba, Parana, Brazil, length, 2 mm; diameter, 1.5 mm) were used. The surface was nanotextured by treating it with a solution consisting of equal volumes of concentrated H_2_SO_4_ and 30% aqueous H_2_O_2_ for 2 h at room temperature under sterile conditions [32].

Laminin-1 (L2020, Sigma-Aldrich, Stockholm, Sweden) was diluted to a concentration of 100 *μ*g/mL in Dulbecco's phosphate-buffered saline (DPBS) without CaCl_2_ or MgCl_2_ (14190-094; GIBCO, Invitrogen Corporation, Grand Island, NY, USA). The implants were subsequently incubated in 48-well plates (Nunclon Surface, Nunc, Roskilde, Denmark) containing 250 *μ*L of the laminin solution per well for 1 h at room temperature. 

To characterize the coated laminin-1, ellipsometry was used in order to estimate the amount of adsorbed laminin-1 on optically smooth titanium surfaces. The descriptive methodology can be found in a study by Linderbäck et al. [33]. In brief, cleaned SiO_2_ surfaces were placed in an evaporation chamber with final pressure below 1 × 10^−8^ Torr. Approximately 200 nm of titanium was physical vapour deposited (PVD) and spontaneously oxidized at room conditions. Thereafter, the prepared surfaces were fixed in the ellipsometric quvette filled with PBS at room temperature. Angles Δ_0_ and Ψ_0_ were measured in three locations with a Rudolph Research AutoEL III ellipsometer operating in a wavelength of 632.8 nm at a 70° angle of incidence. The quvette was emptied and filled with laminin-1 solution and new angles Δ and Ψ were calculated. The thickness of the adsorbed protein was estimated to be 26 Å by using the MacCrackin algorithm [34].

### 2.3. Animals and Implant Surgery

 Nine lop-eared male rabbits (mean body weight, 4.0 kg) were used for the study. One test (laminin-1-coated) implant and one control (noncoated) implant were inserted into the left and right tibial metaphysis, respectively. The animal study was approved by the Malmö/Lund, Sweden regional animal ethics committee (approval number: M282-09). 

 Before surgery, the hind legs were shaved and disinfected with 70% ethanol and 70% chlorhexidine. The animals were anaesthetised with intramuscular injections of a mixture of 0.15 mL/kg medetomidine (1 mg/mL Dormitor; Orion Pharma, Sollentuna, Sweden) and 0.35 mL/kg ketamine hydrochloride (50 mg/mL Ketalar; Pfizer AB, Sollentuna, Sweden). Lidocaine hydrochloride (Xylocaine; AstraZeneca AB, Södertälje, Sweden) was administrated as the local anaesthetic at each insertion site at a dose of 1 mL. After the operation, buprenorphine hydrochloride (0.5 mL Temgesic; Reckitt Benckiser, Slough, UK) was administered as an analgesic for 3 days. After 2 weeks, the rabbits were sacrificed with an overdose (60 mg/mL) of pentobarbital natrium (Apoteksbolaget AB, Stockholm, Sweden). 

### 2.4. Observation of the Implant Interface by SEM

Implants from both groups (*n* = 3) were removed from the tibiae, cleaned in 4% neutral-buffered formaldehyde solution for 10 min, dehydrated using an ascending series of ethanol, and dried. The retrieved implant samples were observed using SEM under various magnifications.

### 2.5. Extraction of RNA and Real-Time RT-PCR

For gene expression analysis, both control and test groups from all 9 rabbits were removed and the retrieved samples were placed in RNA later solution (QIAGEN GmbH, Hilden, Germany) until analysis. In order to obtain detectable RNA, each of the 9 samples in the control and test groups were pooled for RNA isolation. Total RNA was isolated from the surrounding tissue using Trizol reagent (Gibco BRL, Carlsbad, CA, USA) and EZNA tissue RNA isolation kit (Omega Bio-Tek, Norcross, GA, USA). Total RNA was quantified using a nanodrop spectrophotometer (Thermo Scientific NanoDrop Technologies, Wilmington, DE). 

The reverse transcription reaction test was performed according to the manufacturer's instructions, using the high capacity cDNA archive kit (Applied Biosystems, Foster City, USA). 600 ng total RNA was mixed with 100 uL reaction volume of reverse transcriptase (RT) buffer, primers, nuclease-free water, and MultiScribe RT.

Real-time quantitative PCR was conducted under standard enzyme and cycling conditions on a StepOne system, using custom-designed real-time assays and SYBR green detection (PrimerDesign Ltd., Southampton, UK) (Table 1). cDNA corresponding to 6 ng of mRNA was used in each PCR reaction, and mixtures were prepared according to the manufacturer's instructions in 10 *μ*L triplicates for each target cDNA. Amplification was carried out in 96-well thermal cycle plates on a StepOne system (Applied Biosystems). The data were analysed using a comparative Ct method by StepOne. Gene expression levels were normalized with the housekeeping gene *β*-actin. Glyceraldehyde-3-phosphate dehydrogenase (GAPDH) served as an endogenous control.

## 3. Results

### 3.1. Implant Characterization

**Figure 1:**
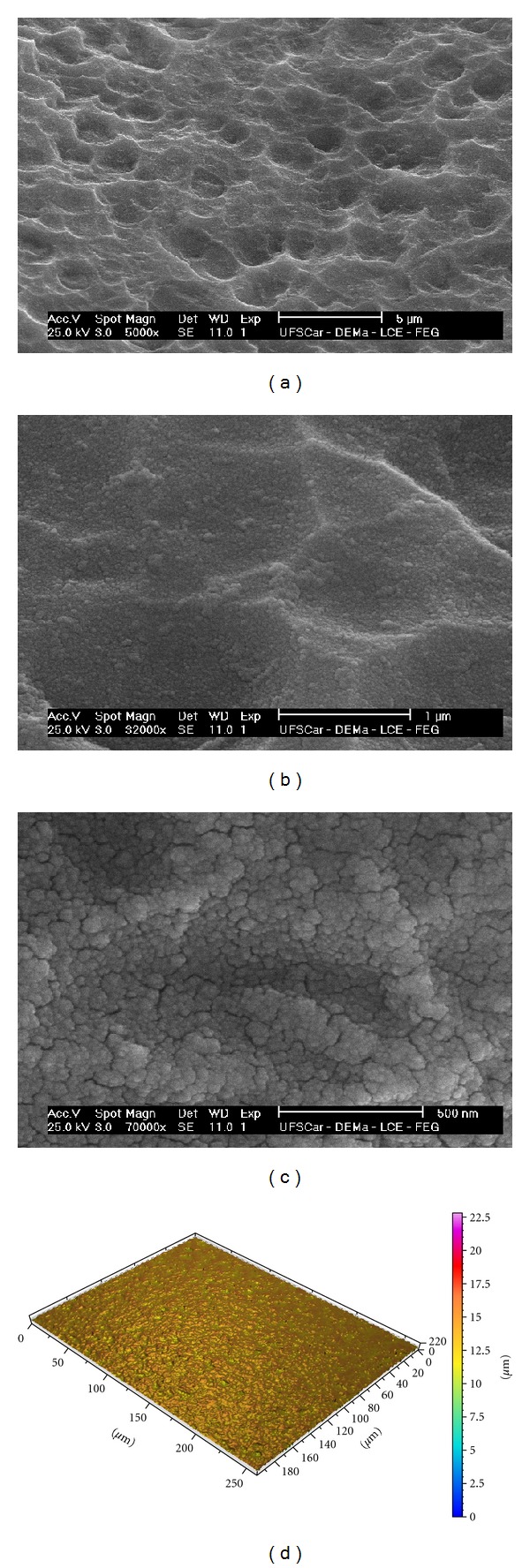
SEM micrographs of nanoroughened implant surface before protein coating (magnification (a) ×5,000, (b) ×32,000, and (c) ×70,000). (d) Interferometer image of nanoroughened implant surface before protein coating (measurement area: 260 mm × 200 mm).

The SEM image for the surface of the nanostructures is presented in Figures 1(a)–1(c), which depicted homogeneous nanostructures covering the entire implant surface. Nanostructures below 50 nm were identified at higher magnification images (Figure 1(c)).

The mean *S*_*a*_ ± (SD) was 0.28 ± (0.07) *μ*m; *S*_ds_ ± (SD) was 195,203 (7,871); *S*_dr_ ± (SD) was 8.15 (0.53)%.  Figure 1(d) shows three-dimensional optical interferometry image of the surface.

### 3.2. Scanning Electron Microscopic Observation of the Retrieved Implants

**Figure 2:**
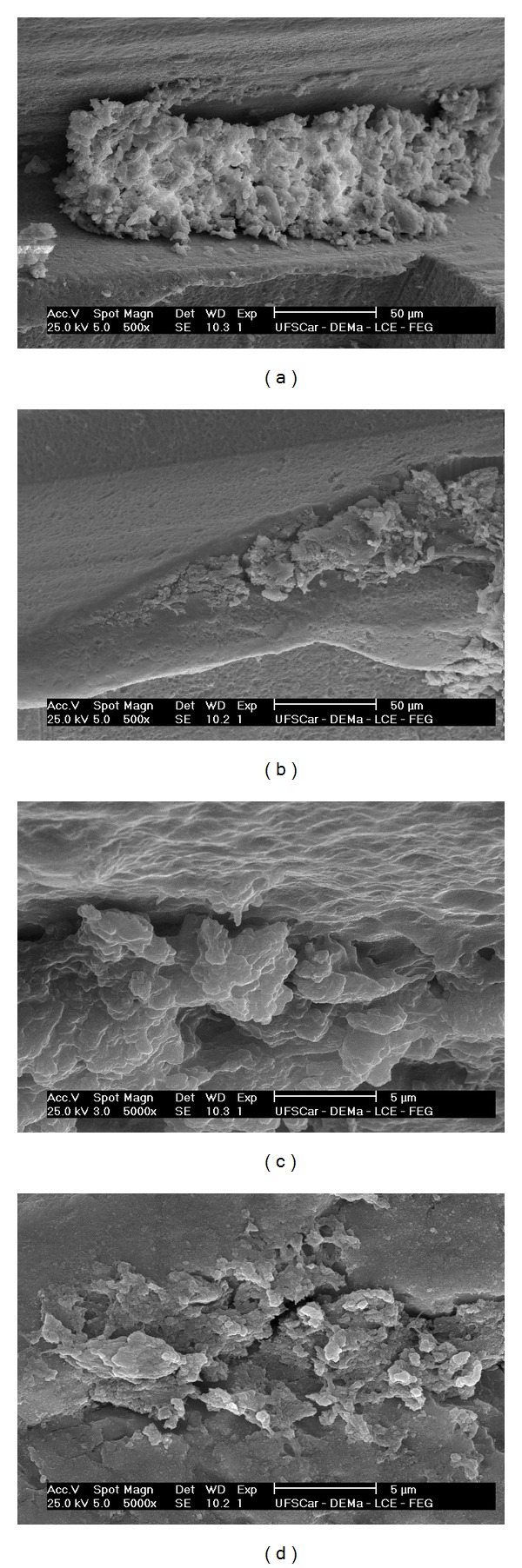
Scanning electron micrographs of retrieved implants for (a) nanoroughened implant surface (control), (b) nanoroughened implant surface + laminin-1 (test) (magnification ×500), (c) higher magnification of the nanoroughened implant surface (control) (×5000), and (d) higher magnification of the nanoroughened implant surface + laminin-1 (test) (×5000).

Out of the 3 samples of each group, Figures 2(a)–2(d) present representative electron micrographs for the samples that remained for 2 weeks *in vivo*. In both control and test implants, remnants of some bony tissue were visible. No remarkable morphologic and quantitative differences were observed between the 2 groups. 

### 3.3. Gene Expression

**Figure 3:**
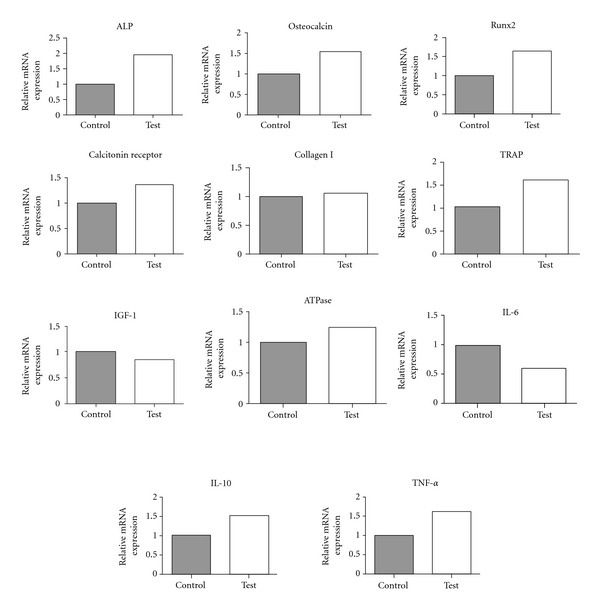
Gene expressions of bone formation markers by real-time RT-PCR for the non-laminin-1-coated (control) and -coated (test) groups. After 2 weeks, the surrounding tissues of implants were collected and total RNA of pooled samples was isolated. The osteogenic markers (ALP, osteocalcin, Runx2, calcitonin receptor, collagen I, TRAP, IGF-1, and ATPase) and inflammation markers (IL-6, IL-10, and TNF-*α*) were evaluated and higher values were detected for the experimental group. The relative expressions of target genes were normalized with housekeeping gene *β*-actin.

The results of real-time RT-PCR are presented in Figure 3. In general, the osteoblast markers that presented higher expression in the case of the test implants were RUNX-2 (1.62-fold), osteoclcin (1.53-fold), alkaline phosphatase (ALP) (1.97-fold), and collagen I (1.04-fold). On the other hand, the expression of IGF-1 was low (0.84-fold). In the case of the test implants, all osteoclast markers investigated in the present study showed higher expression for the experimental group relative to control, namely, tartrate-resistant acid phosphatase (TRAP) (1.67-fold), calcitonin receptor (1.35-fold), and ATPase (1.25-fold). The inflammatory markers that showed higher expression for the test implants than the control implants were IL-10 (1.53-fold) and TNF-*α* (1.61-fold), whereas IL-6 showed lower expression (0.59-fold).

## 4. Discussion

Protein doping is considered one of the promising methods of surface modifications for hastening the early stages of osseointegration both qualitatively and quantitatively [8, 27, 35, 36]. In most studies concerning protein doping of implant surfaces, the beneficial enhancements were primarily restricted to the initial stages of healing and have been shown to have smaller effects when longer periods of experimental time were observed. Such early effect may be related to competitive protein adsorption, and thereby given the protein adsorption desorption dynamics at the implant surface region, protein doping of implant surfaces is indeed expected to be effective in the initial stages of osseointegration. Such an improvement and upregulation of the early bone response is of great clinical importance since it is in both practitioners' and patients' interest that implants osseointegrate faster for shortening the treatment period.

The results obtained by RT-PCR showed distinct differences between the nanostructured surface with/without laminin-1-coating. Evaluation of the selected osteoblastogenesis-related markers revealed that most of the markers showed higher expression around laminin-1-coated implants relative to the control implant group. It is of great interest that the expression levels of ALP, RUNX-2, and collagen I were higher in the case of the laminin-1-coated implant since these markers are indicators of higher osteoprogenitor and osteoblast precursor activity [37]. In addition, the higher expression of osteocalcin, the specific marker for bone formation, indicates that the differentiation activity of cells into osteoblasts was upregulated around laminin-1-coated implants [38]. On the other hand, lower amounts of IGF-1 expression (reported to promote osteoblast activity [39] and osteoblast proliferation [40]) were detected around laminin-1-coated implants, indicating that the proliferation activity at the interface was suppressed. While our results are contradictory concerning the early osteogenic events, osteoblast proliferation and differentiation activity have been previously reported to be contradictory [41, 42]. Thus, the doped laminin may have suppressed proliferation while upregulating differentiation. 

Osteoclast-mediated bone resorption around dental implants plays an important role in bone remodelling and thereby osseointegration establishment and maintenance [43, 44]. In the present study, all osteoclastic markers tested presented higher expression for the laminin-1-coated implant. It has been reported that osteoblastogenesis and osteoclastogenesis transact and regulate each other through the receptor activator of nuclear factor-kappa B ligand (RANKL)/RANK/osteoprotegerin (OPG) system pathway [45, 46]. Thus, we speculate that the higher level of osteoblastic gene expression may have induced higher osteoclastic gene expression. In the present study, the osteoclastic activity may have been highly active due to mutual interactions after surface doping with laminin. It is well recognized that integrin *α*_*V*_ *β*_3_, which is highly produced by osteoclasts, presents high affinity for the RGD-motif, which is included in many of the extracellular matrix proteins. Although this might be a non-laminin specific mechanism, another integrin molecule, that is, *α*_2_ *β*_1_, is also expressed by mammalian osteoclasts and is highly specific for laminin and collagen. Thus, the increase in osteoclast proliferation denoted by higher levels of TRAP and calcitonin receptor may be laminin specific directly by means of laminin/*α*_2_ *β*_1_ interaction [47]. 

The RT-PCR results also demonstrated that most of the inflammatory factors were upregulated for the laminin-1-doped group. Haapasalmi et al. have reported that laminin-1 localises where inflammation exists, as seen in chronic periodontal inflammatory responses [48]. Since inflammatory reactions are part of the healing process [49], the induced inflammatory gene expression further supports higher degrees of osteogenesis at the laminin-1-coated implant interface. For example, TNF-*α* has been proven to be necessary for intramembranous ossification [50] and to increase matrix mineralization and the levels of bone morphogenic protein-2 and alkaline phosphatase *in vitro* [51]. These findings are in agreement with the observed increase of osteoclcin and alkaline phosphatase in our study.

A study in knockout IL-10 mice has demonstrated decreased gene expression of alkaline phosphatase and osteocalcin in the absence of the IL-10 gene [52]. Thus, the elevated levels of IL-10 in our study are well correlated to the increased gene expression of those osteoblast markers. Additionally, IL-6 has been reported to stimulate osteoclastic bone resorption [53] hence explaining the enhanced expression of osteoclastic markers.

The SEM investigation depicted similar bone formation on both surfaces. It is speculated that because of the early time point, the mechanical attachment strength of the bone tissue to the implant surface may be low, and the mineralisation is still in progress. This statement is further supported by the high gene expression of ALP, which indicates that bone is still under maturation. For this reason, it is potentially like that large segment of immature bony tissues may have detached from the implant interface, rather than development of a fracture within the bone. 

Although the results of this study are preliminary, the information motivates further investigation of the novel protein we utilized in the current study as an implant coating. Further, evaluation of gene expression may help capture detailed differences, which may be difficult to detect with the conventional imaging and biomechanical evaluation techniques. 

## 5. Conclusion

We hypothesised that the addition of laminin-1 would enhance osteogenic markers in the early stages of osseointegration. Compared to the noncoated nanostructured implant surface, the protein-doped nanostructured implant surface presented higher gene expression of typical genes involved in the osseointegration cascade, and therefore the hypothesis of the study was accepted.

## Figures and Tables

**Table 1:** Primers used and specific parameters of the real-time PCR.

Gene	Primer sequence	Tm	Amplicon size (bp)	Primer source
ALP	S TGGACCTCGTGGACATCTG	75	80	Oryctolagus cuniculus
	A CAGGAGTTCAGTGCGGTTC			
ATPase	S CCTGGCTATTGGCTGTTACG	77.7	98	Oryctolagus cuniculus
	A GCTGGTAGAAGGACACTCTTG			
Calcitonin receptor	S CGTTCACTCCTGAAAACTACA	72.6	128	Oryctolagus cuniculus
	A GCAACCAAGACTAATGAAACA			
Collagen I	S GGAAACGATGGTGCTACTGG	80.4	83	Oryctolagus cuniculus
	A CCGACAGCTCCAGGGAAG			
IGF-1	S CCGACATGCCCAAGACTCA	70.3	81	Oryctolagus cuniculus
	A TACTTCCTTTCCTTCTCCTCTGA			
IL-6	S GAGGAAAGAGATGTGTGACCAT	73.5	104	Oryctolagus cuniculus
	A AGCATCCGTCTTCTTCTATCAG			
IL-10	S CCGACTGAGGCTTCCATTCC	73.3	75	Oryctolagus cuniculus
	A CAGAGGGTAAGAGGGAGCT			
Osteocalcin	S GCTCAHCCTTCGTGTCCAAG	77.8	70	Oryctolagus cuniculus
	A CCGTCGATCAGTTGGCGC			
Runx2	S GCAGTTCCCAAGCATTTCATC	72.8	81	Oryctolagus cuniculus
	A GTGTAAGTAAAGGTGGCTGGATA			
TNF-*α*	S CTCACTACTCCCAGGTTCTCT	78.2	122	Oryctolagus cuniculus
	A TTGATGGCAGAGAGGAGGTT			
TRAP	S GCTACCTCCGCTTCCACTA	78.5	129	Oryctolagus cuniculus
	A GCAGCCTGGTCTTGAAGAG			
*β*-actin	S CACCCTGATGCTCAAGTACC	76.4	96	Oryctolagus cuniculus
	A CGCAGCTCGTTGTAGAAGG			
